# Long-term survival of patients with central or > 7 cm T4 N0/1 M0 non-small-cell lung cancer treated with definitive concurrent radiochemotherapy in comparison to trimodality treatment

**DOI:** 10.1186/s13014-022-02080-9

**Published:** 2022-07-16

**Authors:** Nika Guberina, Christoph Pöttgen, Martin Schuler, Maja Guberina, Georgios Stamatis, Till Plönes, Martin Metzenmacher, Dirk Theegarten, Thomas Gauler, Kaid Darwiche, Clemens Aigner, Wilfried E. E. Eberhardt, Martin Stuschke

**Affiliations:** 1 Department of Radiation Therapy, West German Cancer Center (WTZ), University Hospital Essen, Hufelandstraße 55, 45147 Essen, Germany; 2 Department of Medical Oncology, West German Cancer Center, University Hospital Essen, Essen, Germany; 3 Division of Thoracic Oncology, West German Cancer Center, University Medicine Essen - Ruhrlandklinik, Essen, Germany; 4 German Cancer Consortium (DKTK), Partner Site University Hospital Essen, Essen, Germany; 5 Department of Thoracic Surgery, West German Cancer Center, University Medicine Essen - Ruhrlandklinik, Essen, Germany; 6 Institute of Pathology, University Hospital Essen, Essen, Germany; 7 Department of Pulmonary Medicine, West German Cancer Center, Section of Interventional Pneumology, University Medicine Essen - Ruhrlandklinik, Essen, Germany

**Keywords:** Definitive radiochemotherapy, Non-small cell lung cancer, TNM-staging

## Abstract

**Background:**

To examine long-term-survival of cT4 cN0/1 cM0 non-small-cell lung carcinoma (NSCLC) patients undergoing definitive radiochemotherapy (_cc_RTx/CTx) in comparison to the trimodality treatment, neoadjuvant radiochemotherapy followed by surgery, at a high volume lung cancer center.

**Methods:**

All consecutive patients with histopathologically confirmed NSCLC (cT4 cN0/1 cM0) with a curative-intent-to-treat _cc_RTx/CTx were included between 01.01.2001 and 01.07.2019. Mediastinal involvement was excluded by systematic EBUS-TBNA or mediastinoscopy. Following updated T4-stage-defining-criteria initial staging was reassessed by an expert-radiologist according to UICC-guidelines [8th edition]. Outcomes were compared with previously reported results from patients of the same institution with identical inclusion criteria, who had been treated with neoadjuvant radiochemotherapy and resection. Factors for treatment selection were documented. Endpoints were overall-survival (OS), progression-free-survival (PFS), and cumulative incidences of isolated loco-regional failures, distant metastases, secondary tumors as well as non-cancer deaths within the first year.

**Results:**

Altogether 46 consecutive patients with histopathologically confirmed NSCLC cT4 cN0/1 cM0 [cN0 in 34 and cN1 in 12 cases] underwent _cc_RTx/CTx after induction chemotherapy (_i_CTx). Median follow-up was 133 months. OS-rates at 3-, 5-, and 7-years were 74.9%, 57.4%, and 57.4%, respectively. Absolute OS-rate of _cc_RTx/CTx at 5 years were within 10% of the trimodality treatment reference group (Log-Rank *p* = 0.184). The cumulative incidence of loco-regional relapse was higher after _i_CTx + _cc_RT/CTx (15.2% vs. 0% at 3 years, *p* = 0.0012, Gray’s test) while non-cancer deaths in the first year were lower than in the trimodality reference group (0% vs 9.1%, *p* = 0.0360, Gray’s test). None of the multiple recorded prognostic parameters were significantly associated with survival after _i_CTx + _cc_RT/CTx: Propensity score weighting for adjustment of prognostic factors between _i_CTx + _cc_RT/CTx and trimodality treatment did not change the results of the comparisons.

**Conclusions:**

Patients with cT4 N0/1 M0 NSCLC have comparable OS with _cc_RTx/CTx and trimodality treatment. Loco-regional relapses were higher and non-cancer related deaths lower with _cc_RTx/CTx. Definitive radiochemotherapy is an adequate alternative for patients with an increased risk of surgery-related morbidity.

**Supplementary Information:**

The online version contains supplementary material available at 10.1186/s13014-022-02080-9.

## Background

The majority of locally advanced lung tumours with invasion of central anatomic areas such as mediastinum or large vessels often present with synchronous lymph nodal metastasis at initial diagnosis. However, cT4 cN0/1 cM0 lung cancer amounts about one fourth of stage III NSCLC according to the 8th edition of the TNM classification in the International Association for the Study of Lung Cancer database and therefore is a major subgroup [1, 2]. The focus of this analysis was laid on this genuine entity, where surgery is conventionally accepted as principle curative-intended treatment modality. T4 lymph nodal negative tumors are regarded as a subset with prognostically better outcome than T4 lymph nodal positive tumors [1–5], which is believed to be owed to a lower metastatic propensity. Contrary to the whole group of unresectable stage III non-small cell lung tumors, where definitive radiochemotherapy played even prior to the practice changing PACIFIC trial a major role [6, 7], in T4 nodal negative lung tumors definitive radiochemotherapy represents only another treatment option [8]. Studies, defining outcomes after definitive radiochemotherapy in the clinical T4 N0 M0 category after mediastinoscopy or EBUS-TBNA for all patients and compare data retrospectively with trimodality treatment are rather limited [9]. Therefore, we decided to conduct this analysis on patients with clear T4-defining criteria (8th edition UICC) and exclusion of mediastinal lymph node involvement according to the initial staging with EBUS-TBNA or mediastinoscopy and to consider all consecutive patients that underwent definitive radiochemotherapy in a curative-intention to treat. The purpose of this study was to examine long-term overall and progression-free survival of T4 N0/1 M0 NSCLC patients undergoing definitive concurrent radiochemotherapy (_cc_RTx/CTx) in comparison to the trimodality treatment neoadjuvant radiochemotherapy followed by surgery that was the preferred treatment option outside clinical trials for patients not selected for up-front surgery at a high volume lung cancer center in the considered time periods. In addition, a competing risks analysis was performed to compare cumulative incidences of isolated loco-regional recurrences as first site of relapse, distant metastases, secondary tumors and non-cancer deaths after definitive radiochemotherapy or trimodality treatment for this group of patients. For intermodality comparison the identical eligibility criteria concerning staging procedures for T4 N0/1 status were used in the present as in the preceding study [10].

## Methods

Approval of the local ethics committee was obtained prior to data collection and analysis [21–10,203-BO]. Furthermore, the research was conducted following the statutes of the Declaration of Helsinki 1964. In a retrospective study design all consecutive patients with confirmed NSCLC T4 N0-1 M0 [stage III] treated in the period from 01.01.2001 to 01.07.2019 with definitive, concurrent curative-intent-to-treat radiochemotherapy (_cc_RTx/CTx) at a high volume cancer center were included. Altogether 21 patients in that time period were treated within the ESPATUE trial, a randomized, prospective investigator initiated trial (Phase III study of surgery versus definitive concurrent radiochemotherapy boost in patients with resectable stage IIIA (N2) and selected IIIB non-small-cell lung cancer after induction chemotherapy and concurrent radiochemotherapy) [11]. Results from this study were compared with data from this group on consecutive T4 N0-1 M0 NSCLC patients treated with a trimodality treatment option, induction chemotherapy and neoadjuvant radiochemotherapy followed by surgery [10].

### Tumor and mediastinal staging

Initial staging included computed-tomography (CT) and [^18^F]FDG positron-emission-tomography/computed-tomography (PET/CT) as well as cranial magnet resonance imaging. In order to exclude mediastinal lymph node involvement systematic endobronchial ultrasound-guided transbronchial needle aspiration (EBUS-TBNA) or mediastinoscopy was conducted. Prior to treatment initiation each patient case was demonstrated and discussed in an interdisciplinary tumor board and patients with potentially resectable tumors were discussed again in the tumor board during the week prior approaching a cumulative radiation dose of 45 Gy on the basis of all clinical data (patient history, performance status, lung function and cardiac examination, lung perfusion, present CT or [^18^F]FDG PET/CT). T4 defining criteria at initial diagnosis were reassessed by an expert-radiologist according to updated UICC-guidelines [8th edition]. Meanwhile, the assessment of infiltration of important anatomical structures in imaging may be challenging with differing final pathologic findings [12–14]. Hence, we relied on strict criteria of infiltration, a circumferential encasement of more than 180°, endovascular or endoluminal tumor growth, large invasion or osteolytic destruction. Furthermore, the cavitation diameter/tumor diameter ratio (CTR) was analyzed to identify tumor configurations with a higher risk of fatal pulmonary hemorrhage and infection according to the criteria by Ito et al. [15].

### Treatment sequence

With special regard to the performance status induction chemotherapy (_i_CTx) was administered prior to definitive radiochemotherapy (_cc_RTx/CTx). If the clinical performance status was adequate, concurrent cisplatin- and vinorelbine based chemotherapy was administered d1 and d8 (two cycles). Alternative chemotherapy regimens included cisplatin or carboplatin weekly, concurrent carboplatin and vinorelbine or cisplatin and etoposide. Since 21.09.2018, when durvalumab (IMFINZI®; September 2018) was granted marketing authorization in the European Union, patients with appropriate inclusion criteria received consolidation immunotherapy after _cc_RTx/CTx. No patient participated in the Expanded-Access-Program EAP which lasted from 22.11.2017—15.10.2018 [16]. Table 1 sums up patients’ characteristics.

**Table 1:** Highlighting individual patient characteristics (age; gender) including initial diagnosis (histology; TNM-stage disease, tumour features)

	Absolute n	Percentage %
*Age years*		
< 60 years	20	43.4
≥ 60 years	26	56.6
*Gender*		
Female	12	26.1
Male	34	73.9
*Histology*		
Adenocarcinoma	12	26.1
Squamous-cell carcinoma	33	71.7
Non other specified	1	2.2
*TNM-stage disease*		
T4 N0 M0	34	73.9
T4 N1 M0	12	26.1

Clinical performance status	Absolute n	Percentage %
*ECOG-Status*		
ECOG 0	27	58.7
ECOG 1	18	39.1
ECOG 2	1	2.2
*NYHA-Class*		
No cardiac insufficiency	32	69.6
NYHA 1	10	21.7
NYHA 2	4	8.7
*COPD-Score (GOLD)*		
No pulmonary obstruction	17	36.9
COPD 1	5	10.9
COPD 2	20	43.5
COPD 3	3	6.5
COPD 4	1	2.2

	Absolute n	Percentage %
Operable	17	36.9
Functional non-operable	25	54.4
Technical non-operable	4	8.7
Total	46 pts	100%

Tumor characteristics	Mean	Range
Grading	2.5	1–4
PD-L1 [%]	27.25 [12 pts tested]	0–100
Post-CTx MTV	61.7 [29 pts]	0.2–265.2
SUV_max_	4.1	2.0–13.8

### Radiotherapy

The radiation technique/ modality has changed over time. Before 2012, 3D conformal radiotherapy was used as the main modality. Since 2012, static field IMRT has been the most widely used technique, which has been gradually replaced by VMAT since 2018. The 3D technique and target volume definition was performed as described for the ESPATUE trial by our group [11]. After January 2013, target volume definition from the RTOG 617 trial was adopted. Total radiation doses ranged from 60 to 71 Gy, either conventionally fractionated at 2 Gy per daily fraction, 5 fractions a week, or using hyperfractionated acceleration at 2 × 1.5 Gy/F per day at minimum intervals of 6 h, on 5 days per week up to a total dose of 45 Gy, followed by conventional fractionation. 6 to 8 MeV photons from a linear accelerator were used.

### Endpoints of this study

Endpoints of this study were overall survival (OS) and progression-free survival, as well as loco-regional recurrences, distant metastases, secondary tumors and non-cancer deaths. We predefined a 10% absolute difference in overall survival at 5 years between treatment groups as a boundary to announce differences between arms independent from the outcome of statistical test between survival curves. This boundary was adapted to the precision of the 5 year survival estimates as it amounts about 1.7 the standard error of these estimates [10]. Chemo- and radiotoxicity were assessed following Common Terminology Criteria for Adverse Events (CTCAE) by focusing on grades 3 to 5. Adverse events which were severe but not immediately life-threatening and required hospitalization were regarded as grade 3, while events which were life-threatening and needed intensive care unit admission were assigned to grade 4, and events which led to death represented grade 5.

### Statistics

Descriptive statistics and statistical analysis were executed by means of SAS (version 14.3, SAS Institute, Cary, NC, US) and SPSS, version 27.0 (IBM Corp., Armonk, New York, USA). Overall survival was defined as time from start of radiochemotherapy to death of any cause. Failure time was calculated from start of radiochemotherapy to the date of proven failure, with censoring at last follow up with a chest CT for patients without an event. Failures were isolated loco-regional recurrences, distant metastases as a component of first event, and deaths without relapse. Non-parametric survivor function estimation was performed according the Kaplan–Meier Method (Proc Lifetest, SAS). Log-rank test was used to assess differences between the definitive _cc_RTx/CTX regimen and the trimodality treatment published in [10]. In addition, a competing risks analysis was performed, with isolated loco-regional recurrences, distant metastases as a component of first event, secondary tumors and non-lung cancer deaths were competing risks. Cumulative incidence functions for failures of a specific cause were compared for the definitive _cc_RT/CTx and trimodality groups of patients by the Gray’s test using Proc Lifetest.

Propensity score weighting was used to balance treatment groups according to patient and tumor characteristics between the treatment groups. The inverse probability of treatment weights were used adjusting both treatment groups to the total study group as the standard population. The Cox proportional-hazards model was applied for examining patient and tumor related characteristics as explanatory effects on overall survival or progression free survival.

## Results

Altogether 46 consecutive patients (34 men, 12 women; mean age 62.0 years; range 48.5–81.2 years) with histopathologically confirmed NSCLC cT4 cN0/1 cM0 [cN0 in 34 and cN1 in 12 cases] and with a curative-intent-to-treat treatment sequence were included in a retrospective study design (Table 1). 89.1% of patients underwent [^18^F]FDG-PET/CT for initial staging and exclusion of an M-stage. All patients underwent either systematic EBUS-TBNA including Endoscopic Ultrasound with Bronchoscope-Guided Fine Needle Aspiration (EUS-B) (since 2016) or mediastinoscopy for invasive mediastinal staging (Table 2). T4-stage-defining-criteria were satisfied by at least one criterion, 87% of patients presented with multiple criteria, size (18 cases), mediastinal (38 cases) and great vessel infiltration (39 cases) (Table 3). In 11 cases the main carina, in 27 cases the right or left main bronchus and in 41 cases at least 3 bronchial segments were involved. Altogether, 21 patients were enrolled in the ESPATUE trial for stage IIIA non-small-cell lung cancer and treated in the definitive concurrent-radiochemotherapy (_cc_RTx/CTx) arm [8 deemed non-resectable after a secondary tumor board consensus due to functional reasons, 13 deemed resectable]. From the other 25 patients, 4 patients were deemed resectable, but preferred a definitive _cc_RTx/CTx and 21 patients underwent definitive radiochemotherapy as deemed unresectable due to technical or functional reasons. All 46 patients received induction chemotherapy (_i_CTx) prior to _cc_RTx/CTx. Patients underwent in average 3 cycles of _i_CTx (range 1–5). 43 patients received concurrent cisplatin- and vinorelbine-based _cc_CTx, 1 patient received concurrent cisplatin and etoposide _cc_CTx, 1 patient received concurrent carboplatin and vinorelbine based _cc_CTx and 1 patient started with concurrent cisplatin/ vinorelbine and switched to carboplatin-vinorelbine during the course of radiotherapy due to deterioration of kidney function. Since 21.09.2018, when durvalumab (IMFINZI®; September 2018) was granted marketing authorization in the European Union, 8 patients received consolidation immunotherapy after definitive _cc_RTx/CTx. Twenty nine patients received hyperfractionated accelerated radiotherapy during the first three weeks of radiotherapy, the remaining conventional fractionation. 27 patients received 3D conformal radiotherapy, 8 patients static field IMRT and 11 patients VMAT. Patients and treatment characteristics are given in Tables 1 and 2.

**Table 2:** Summary of diagnostic work-up at initial diagnosis, RTx treatment (target dose, RTx duration, time between start of induction chemotherapy (_i_CTx) and RTx start, mean lung dose, lung V20 and mean heart dose) and sequence of systemic therapy

	Absolute	Percentage (%)
*Staging and clinical work-up*		
Computed Tomography (CT)	46	100.0
[^18^F]FDG positron-emission-tomography/ computed-tomography (PET/CT)	41	89.1
Endobronchial Ultrasound Bronchoscopy (EBUS)/ Endoscopic Ultrasound with Bronchoscope (EUS-B)	19	41.3
Rigid bronchoscopy	46	100.0
Mediastinoscopy	28	60.9

Radiotherapy (RTx)	Mean	Range
Target dose Gy	67.6	60.0–71.0
RTx duration days (approx.)	44.5	31–115
Time between _i_CTx start and RTx start days	79.7	20–150.4
Mean lung dose Gy	14.4	10.4–18.9
Lung V20 %	23.9	6.8–32
Mean heart dose Gy	10.4	1.6–28.6

Systemic agents	Absolute	Percentage (%)
Induction chemotherapy	46	100.0
Concurrent chemotherapy	46	100.0
Adjuvant immunotherapy	8	17.4
Total	46 pts	100%

**Table 3:** T4-defining features on a per patient basis at initial diagnosis assessed on computed tomography, positron emission tomography/ computed tomography and with the help of endobronchial ultrasound: (I) Vertebral body; (II) Diaphragm; (III) Esophagus; (IV) Recurrent laryngeal nerve; (V) Heart; (VI) Trachea; (VII) Carina; (VIII) Tumor size > 7 cm; (IX) Mediastinum; (X) Great vessels

N of patients	Single criterion	Vertebral body	Diaphragm	T4 defining features							
				Esophagus	Recurrent laryngeal nerve	Heart	Trachea	Carina	Tumor size > 7 cm	Mediastinum	Great vessels
Vertebral body	–	–	–	–	–	–	–	–	–	–	–
Diaphragm	–	–	–	–	–	–	–	–	–	–	–
Esophagus	–	–	–	–	–	–	–	–	–	–	–
Recurrent laryngeal nerve	–	–	–	–	1	–	–	–	–	1	1
Heart	1	–	–	–	–	2	–	–	1	1	1
Trachea	–	–	–	–	–	–	4	2	–	4	3
Carina	–	–	–	–	–	–	2	11	2	10	10
Tumor size > 7 cm	–	–	–	–	–	1	–	2	18	17	14
Mediastinum	–	–	–	–	1	1	4	10	17	38	32
Great vessels	5	–	–	–	1	1	3	10	14	32	39

Only 1 primary tumor (2.2%) was cavitated at initial diagnosis with a cavitation/ tumor ratio of 0.4, 7 tumors (15.2%) after _i_CTx with a cavitation/ tumor ratio of mean 0.5 (range 0.3–0.9) and 5 tumors (10.8%) after _cc_RTx/CTx with a cavitation/ tumor ratio of mean 0.3 (0.3–0.4). No patient had a major cavitation/ tumor ratio at the end of radiochemotherapy. No patient suffered fatal pulmonary hemorrhage. The clinical lymph node status was cN0 in 34 and cN1 in 12 patients.

**Fig. 1:**
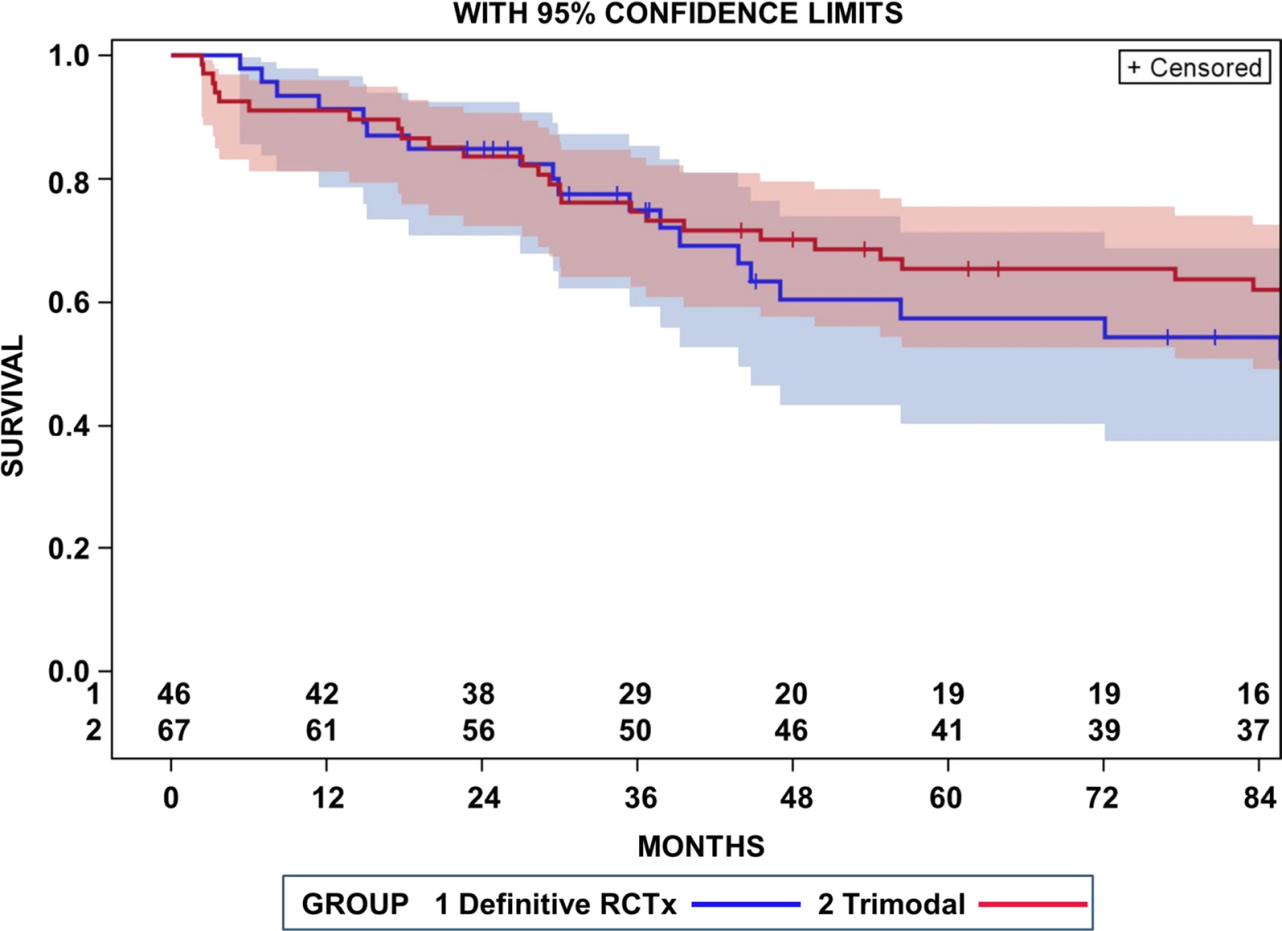
Overall survival of patients with T4 N0/1 M0 non-small cell lung cancer treated with definitive concurrent combination chemotherapy and radiotherapy (RCTx 1). For comparison, survival of NSCLC patients of the same TNM stage who received neoadjuvant radiochemotherapy and surgery were included (trimodal 2). The 95% confidence intervals for an inference at a single fixed time are shown as background areas of the same colour as the respective survivor functions. There were no significant differences between survival curves (*p* = 0.184, Log rank test)

Median follow-up of surviving patients was 61 months, minimum follow-up was 24 months. Overall survival rates in the _cc_RTx/CTx patient group at 2-, 3-, 5-, and 7-years were 84.8%, 74.9%, 57.4%, and 57.4%, respectively. Concerning treatment modality our secondary analyses revealed that OS-rates of _cc_RTx/CTx at 5 years were with within 10% of the trimodality treatment according to our previous analysis (57.4% vs. 65.4%) [10]. Survival curves for patients treated with definitive radiochemotherapy or trimodality were not significantly different [Log Rank (Mantel-Cox) *p* = 0.184] (Fig. 1). Cox proportional-hazards model was performed to explore the association between overall survival and several tumor and patient dependent factors that are summarized in Table 1. In the multivariable proportional hazard analysis using a forward variable selection procedure, none of the factors in Table 1 became significant.

After durvalumab was granted marketing authorization 12/46 patients were tested for PDL1-status (Clone 22C3), 9 positive with a mean PDL1-expression of 29.3% (range 2–100%). A total of 8 patients received durvalumab consolidation therapy.

**Fig. 2:**
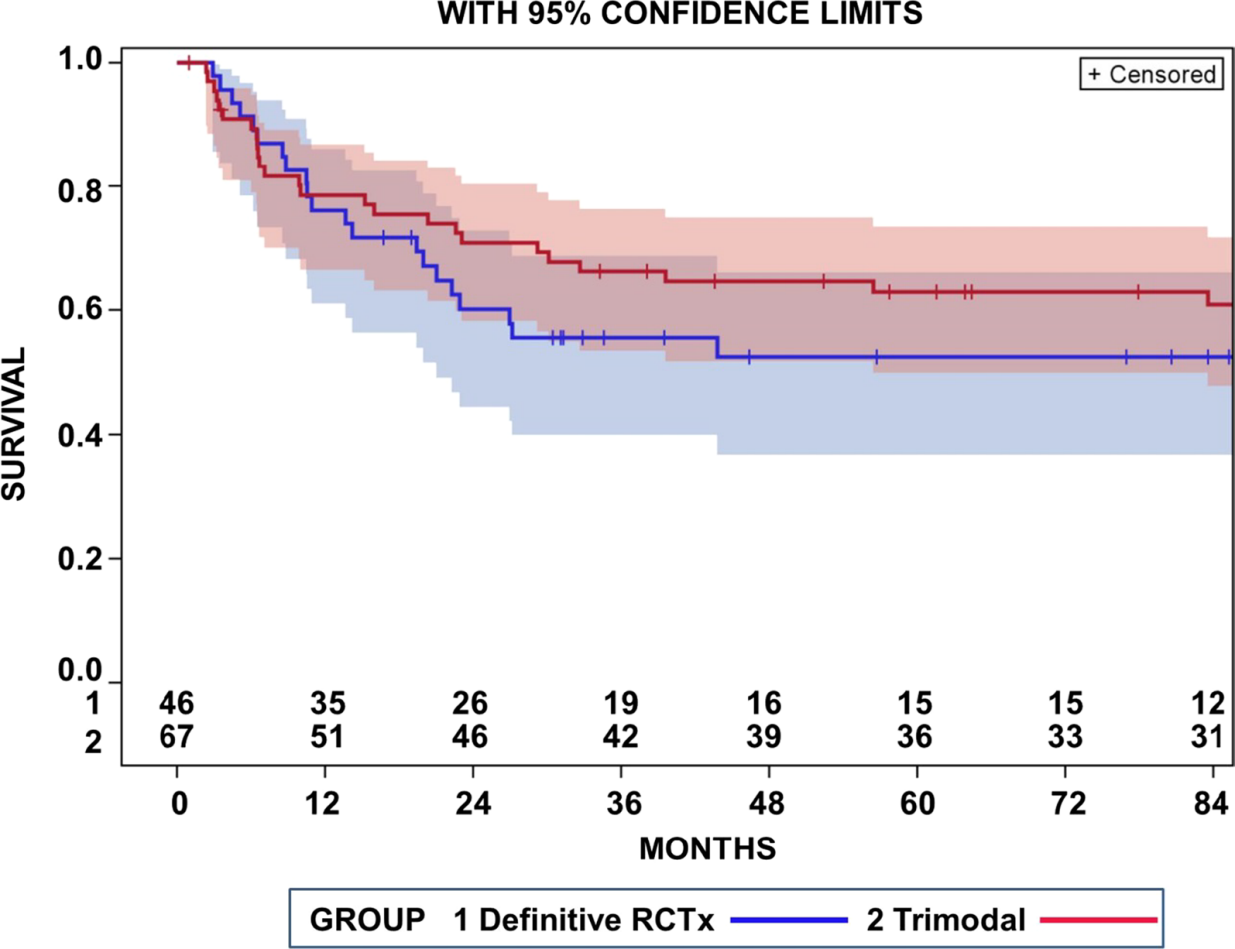
Progression free survival of patients with T4 N0/1 M0 non-small cell lung cancer treated with definitive _cc_RTx/CTx (RCTx 1) in comparison to patients of the same TNM stage who underwent a trimodality treatment (trimodal 2) (*p* = 0.276, Log rank test)

Progression-free-survival-rates at 2-, 3-, 5-, and 7-years were 60.2%, 55.5%, 52.5%, and 52.5% after definitive _cc_RTx/CTx and 70.9%, 66.2%, 62.9%, and 60.9% after trimodality treatment (Fig. 2). The PFS-curves after definitive _cc_RTx/CTx were not significantly different from those of the trimodality treatment (Log rank *p* = 0.276). Progression-free-survival-rates of 50% at 2- and 3-years were similar for the patients treated with durvalumab consolidation and the whole group of patients treated with definitive _cc_RTx/CTx. Comparing patient and tumor characteristics age, gender, histology, and N-category of the definitive irradiated patients with those who had received trimodality treatment described in the preceding publication [10], we observed a tendency to older patients and a slightly differing distribution over the histopathologic subtypes in the present group of patients receiving definitive radiochemotherapy (see Additional file 1: Table 1). Therefore, we balanced the groups according to the histological subtype and age using inverse probability propensity score weighting. No important influence of propensity score weighting on overall survival or progression-free survival was observed (Additional file 1: Supplementary Table 1). After propensity score weighting the 5-year overall survival was 58.9% vs. 64.9% for the definitive _cc_RTx/CTx and trimodality group (Log rank test *p* = 0. 54), while progression-free survival at 5 years was 55.5% vs. 58.6% for the definitive _cc_RTx/CTx and trimodality group, respectively (*p* = 0.94, Log rank test).

**Fig. 3:**
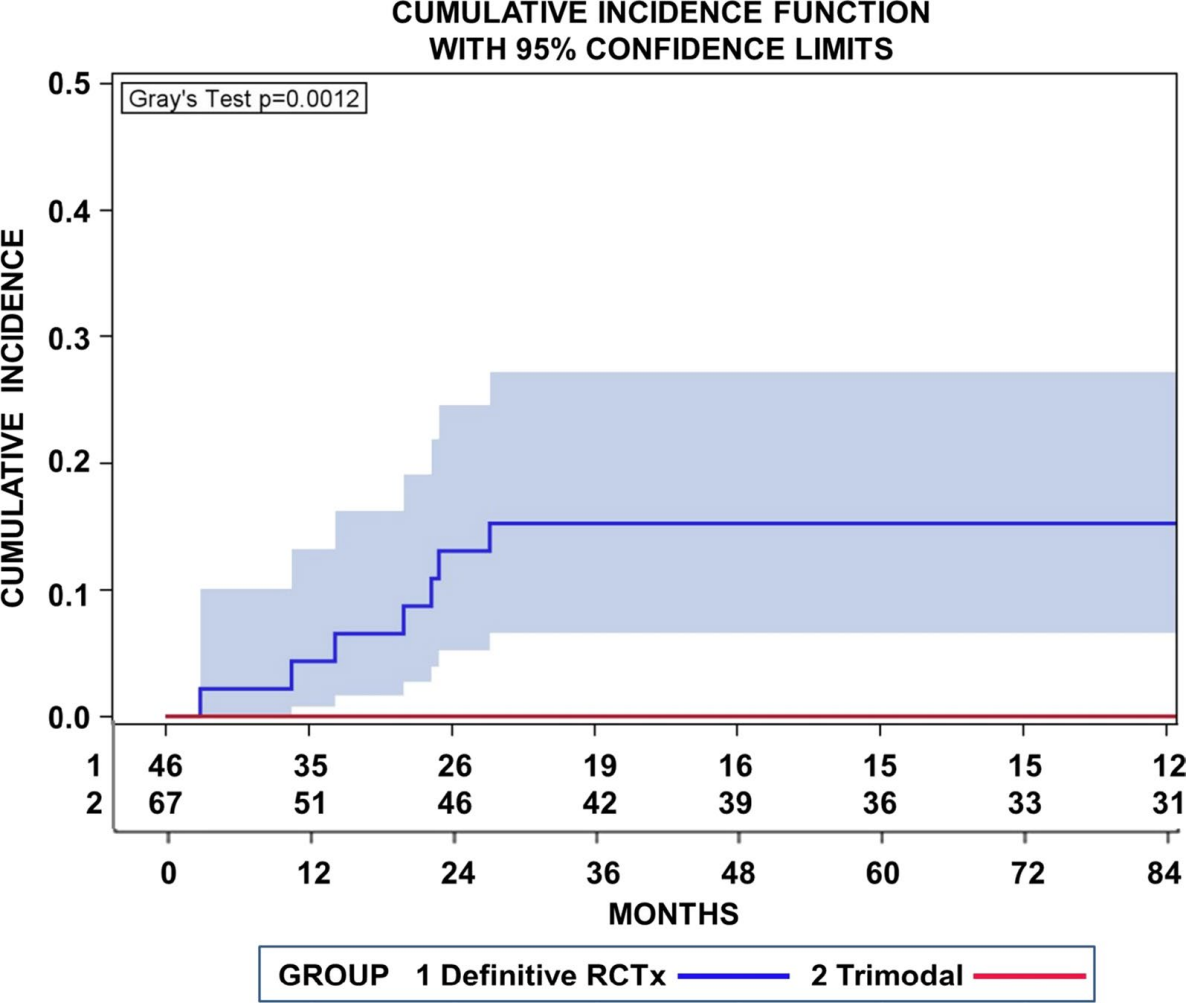
Cumulative incidence function (CIF) for loco-regional recurrences as the first site of relapse for patients treated with definitive radiochemotherapy (RCTx 1) or neoadjuvant radiochemotherapy followed by surgery (trimodal 2). Concurrent risks were distant metastases or secondary cancers. The background areas of the same colour as the CIF represent the pointwise 95% confidence. Also moderate, the incidences of loco-regional recurrences were significantly higher after definitive radiochemotherapy in comparison to trimodality (*p* = 0.0012 Gray’s test)

In addition, we conducted a competing risk analysis, with loco-regional recurrences as first sites of relapse, distant metastases, secondary tumors, and deaths without relapse as concurrent risks. Loco-regional relapses as the first site of relapse were observed in 7 patients after definitive _cc_RTx/CTx. Figure 3 highlights cumulative incidence functions (CIF) for loco-regional recurrences for patients treated with definitive radiochemotherapy or neoadjuvant radiochemotherapy followed by surgery. The cumulative incidence of loco-regional recurrences after definitive _cc_RTx/CTx were significantly higher after definitive radiochemotherapy in comparison to the trimodality treatment (*p* = 0.0012, Gray’s test). The cumulative incidence after definitive _cc_RTx/CTx approached 15.2% (95% CI 6.6–27.2%) at 36 months.

**Fig. 4:**
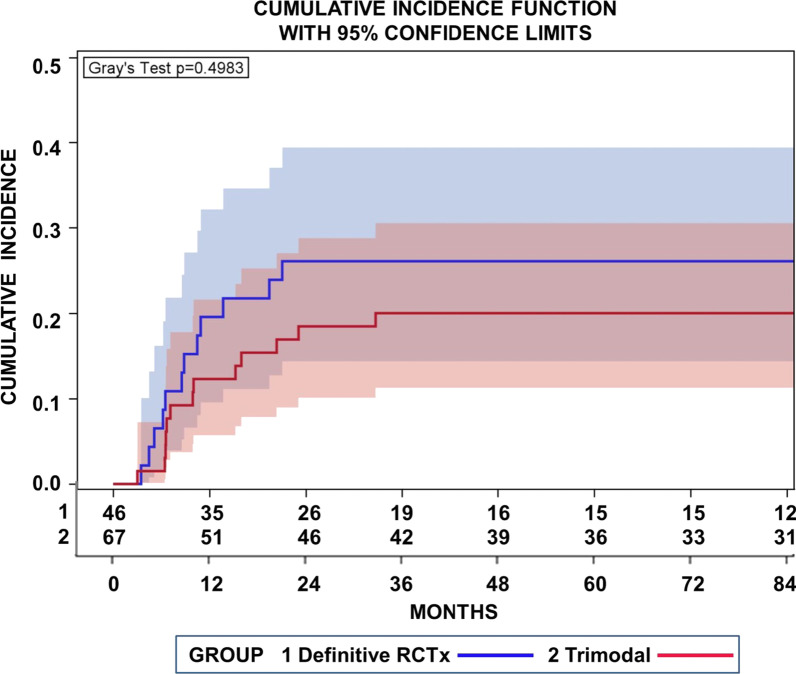
Cumulative incidence function for distant metastases as the first site of relapse for patients treated with definitive radiochemotherapy (RCTx 1) or neoadjuvant radiochemotherapy followed by surgery (trimodal 2). Concurrent risks were local recurrences or secondary cancers. The incidences of distant metastases were not significantly different between definitive radiochemotherapy and the trimodality treatment regimen (*p* = 0.4983 Gray’s test)

**Fig. 5:**
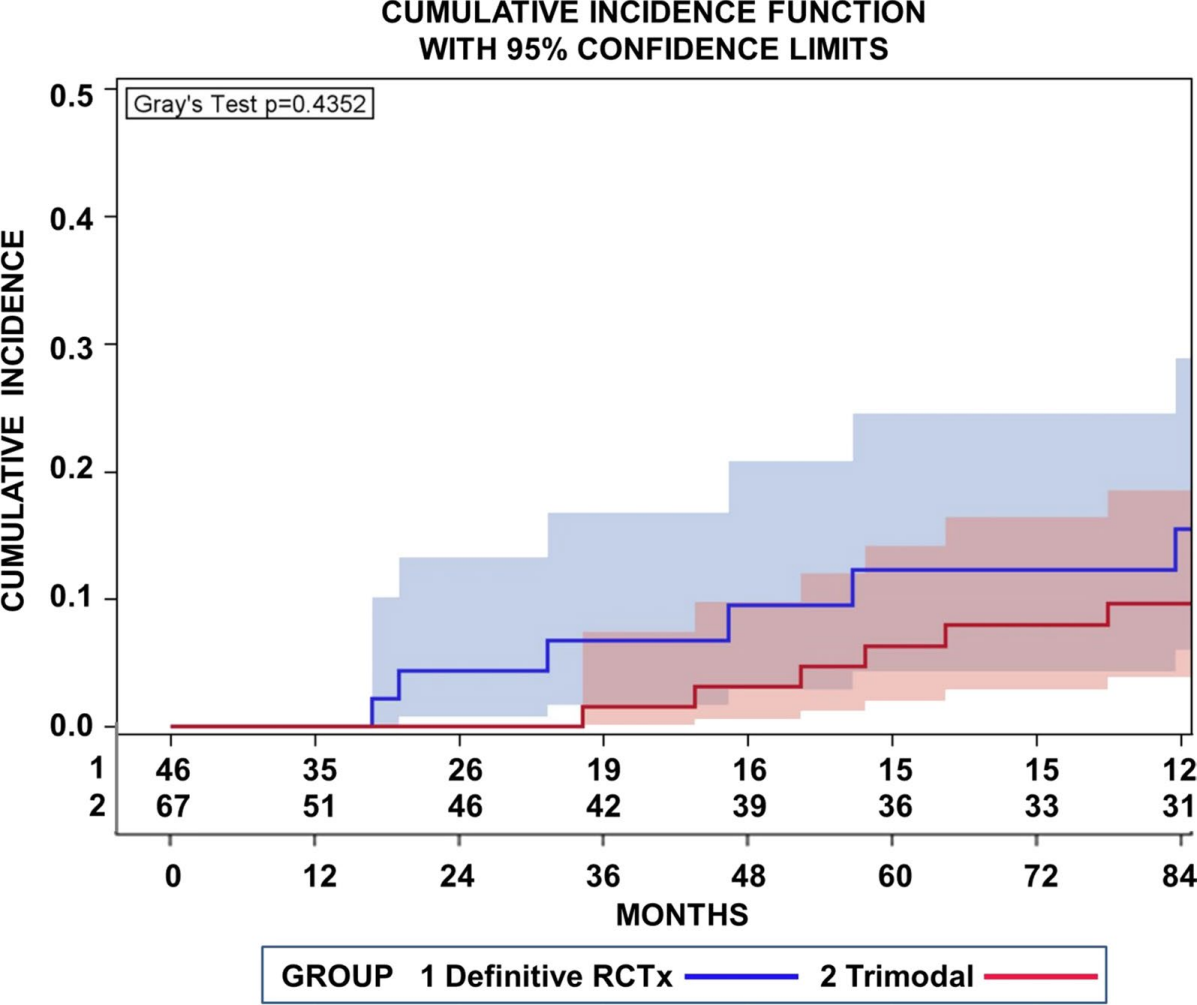
Cumulative incidence function for secondary tumors revealed no significant intermodality difference between incidences of secondary malignanciesfor patients treated with definitive concurrent radiochemotherapy (RCTx 1) compared to those treated with neoadjuvant radiochemotherapy followed by surgery (trimodal 2) (*p* = 0.4352, Gray’s test)

There were no combined distant and local failures as first site of relapse. Distant failures occurred in 12 patients. Metachronous brain metastases occurred in 8 patients. Of these 8 patients 1 patient suffered from combined pancreas and brain metastases, 1 patient from liver metastasis, 1 patient from renal, 1 patient from pleural and 1 patient from pulmonal metastasis. The cumulative incidence of distant metastases approached 26.1% (95% CI 14.4–39.4%) after _cc_RTx/CTx at 24 months and 18.5% (10.1–28.8%) after trimodality treatment. Figure 4 delineates cumulative incidence functions for distant metastases for patients treated with definitive radiochemotherapy or a trimodality treatment schedule. The incidences of distant metastases were not significantly different between definitive radiochemotherapy and the trimodality treatment regimen (*p* = 0.498, Gray’s test). Likewise there was no significant intermodality difference between incidences of secondary malignancies (Fig. 5, *p* = 0.435, Gray’s test). The cumulative incidences of secondary tumors at 84 months approached 15.5% (6.0–28.9%) after definitive _cc_RTx/CTx and 9.6% (3.9–18.6%) after trimodality treatment.

**Fig. 6:**
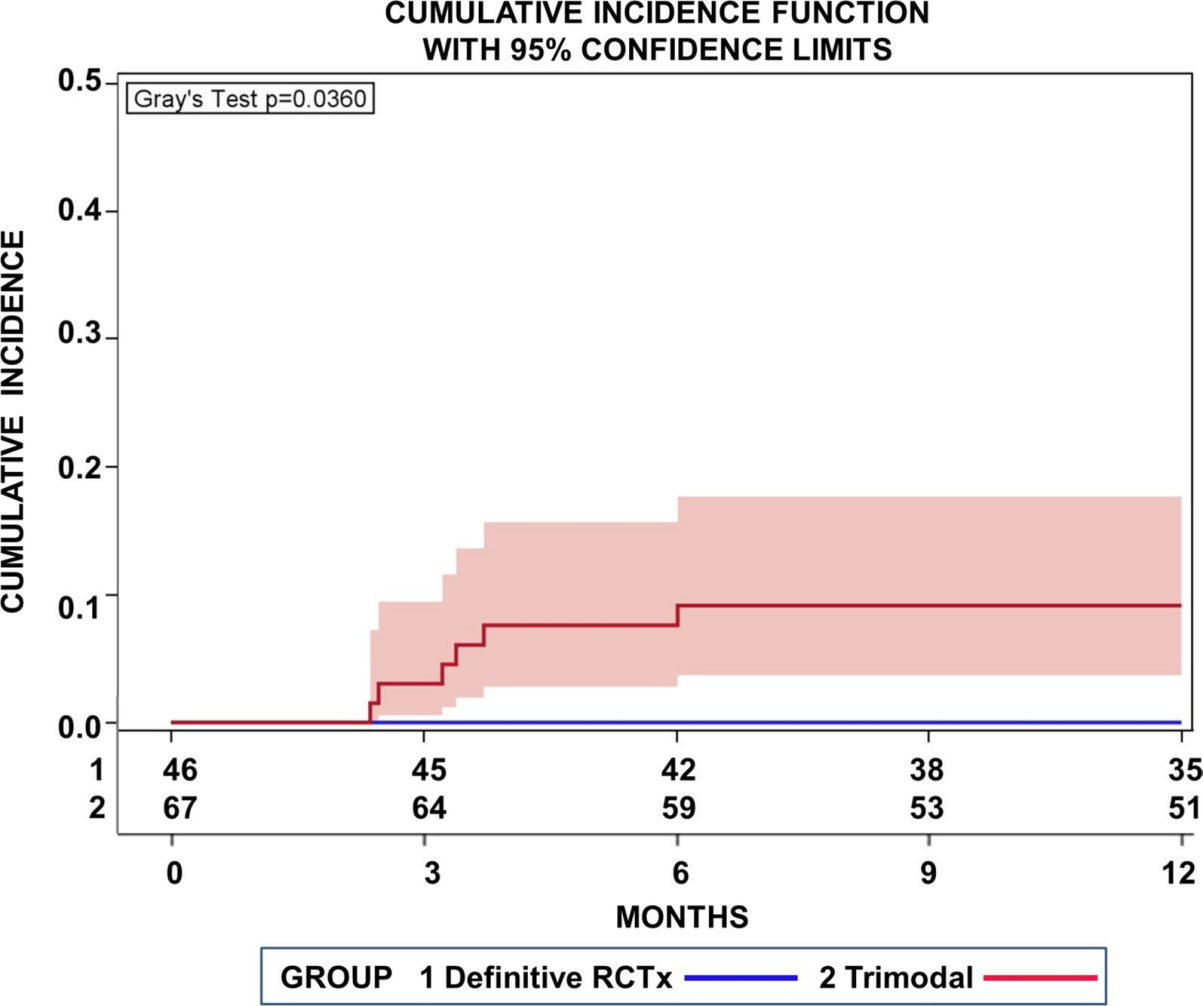
Cumulative incidence function (CIF) for deaths without detected relapse of the NSCLC in the first year. Concurrent risks were deaths after a relapse. The cumulative incidence after surgery was higher indicating a higher post treatment morbidity within the first year for patients treated with definitive radiochemotherapy (RCTx 1) compared to those treated with neoadjuvant radiochemotherapy followed by surgery (trimodal 2) (*p* = 0.0360, Gray’s test)

During _i_CTx 1 patient suffered from grade 4° cytopenia (severe leukopenia), which resulted in an early discontinuation of _i_CTx (1 instead of 3 cycles). 15 patients (32.6%) suffered from CTC AE 2°/3° cytopenia (11 patients from pancytopenia, 3 patients from leukopenia, and 1 patient from anemia). Fatigue, atrial fibrillation and a minor cerebral ischemia which resolved without further neurological deficits were further 2°/3° events emerging during the course of _i_CTx. 12 patients (26.0%) encountered 2°/3° esophagitis and/ or dysphagia during RTx. Figure 6 highlights cumulative incidence functions (CIF) for non-lung cancer deaths without relapse of the NSCLC in the first year. The cumulative incidence of non-lung cancer deaths was higher after surgery and 9.1% (95% CI 3.7–17.7%) after 12 months indicating a higher post treatment morbidity within the first year (*p* = 0.0360, Gray’s test) (Fig. 6).

## Discussion

The reported 5-year survival rates of patients with pathological staged pT4 pN0/1 NSCLC depended on the T4 criterion and ranged different in surgical series from 15% for tumors invading the left atrium to 33% for tumors with invasion of the carina [1]. In a large monoinstitutional series from Paris, up-front surgery resulted in a 5-year survival of 43% for carefully selected pT4 pN0/1 cM0 NSCLC patients, that was better than for patients with pT4 pN2/3 tumors with 17.7% [17]. In the latter group, 9% of patients also had distant metastases. Nonetheless, radical surgery with pneumonectomy should be conducted only in highly skilled lung cancer centers and avoided where possible due to a high morbidity and mortality ranging from 4.6% to 12.5% [18, 19]. Li et al. analyzed clinically staged cT4 cN0/1 M0 tumors from the National Cancer Data Base (NCDB) of the USA based on the size criterion > 7 cm and found a better 5-year survival rate of 42.5% (95% CI 38.1–47.0%) for patients undergoing surgery than for those treated without surgery (13.2% (95% CI 9.1–17.3%)) [20].

Besides radical surgery neoadjuvant radiochemotherapy has been characterized as an important treatment modality to reduce tumor mass, enhancing the R0 resection rate and achieving 5-year overall survival rates in T4 N0/1 M0 up to 65.4% with a median follow-up of 134 months in high-volume cancer centers dedicated to lung cancer treatment [5, 10, 21–25]. De Leyn et al. report a projected 5-year survival rate of 60.3% in 17 patients with centrally located cT4 N0 M0 NSCLC undergoing neoadjuvant radiochemotherapy followed by resection [5]. Long term survival estimates miss due to a short follow-up of 3 years [5]. Overall survival rates reported by Lococo et al. are considerably lower with 41% at 5 years and 0% at 10 years in a collective of 11 patients with cT4 N0 M0, of whom 35% received induction radiochemotherapy [22]. In the prospective phase-II SWOG (Southwest Oncology Group) 8805 study 17 patients with T4 N0/1/X M0 had a median survival time of 28 months undergoing induction radiochemotherapy followed by surgery [23]. Median follow-up was 2.4 years (range, 1.0–3.1). Stupp et al. report a 5-year overall survival rate of 40% in a group of 46 patients with cT1-4 N2/3 M0 and T4 N0/1 M0 NSCLC undergoing neoadjuvant radiochemotherapy followed by surgery, without significant difference between both subgroups [24]. Likewise, Perentes et al. report a 5-year survival rate of 45% in 37 patients with cT4 N0/1 M0 NSCLC and 35 patients with cT4 N2 M0 NSCLC after induction radiochemotherapy followed by resection, also without significant difference between both, prognostically different subsets of subgroups [25]. Results from our group were favourable with an overall 5-year survival of 65.4% that depended on histopathological downstaging and approached 80.5% 5-year survival for the 46% of patients achieving a ypT0 status [10].

Definitive radiochemotherapy in non-resectable T4 N0/1 M0 NSCLC was examined only by a few retrospective reporting 5-year survival rates ranging from 25 to 35% [26, 27]. In the prospective SWOG-9019 study [9] Albain et al. examined survival of patients with cT4 cN0/1 M0 undergoing definitive radiochemotherapy in comparison with the trimodality study SWOG-8805 [23]. Employing identical eligibility criteria as the antecedent comparison trial SWOG-8805 [23] a T4 status was confirmed either by thoracotomy or thoracoscopy, by bronchoscopy with documentation of an involvement of the trachea or carina, by CT, MRI or transesophageal ultrasound with confirmation of a direct invasion of the heart, esophagus, aorta or vertebral body [28]. A N0/1 status required either a negative mediastinoscopy or no mediastinal nodes of any size on CT scan, equivalent to the present study [28]. Albain et al. demonstrated that definitive concurrent radiochemotherapy is feasible with a reasonable toxicity profile. However, median survival for T4 N0/1 M0 disease remained moderate with a median survival of 20 months, and a 5-year survival rate of 17% [9]. These results were inferior to those after trimodality treatment from SWOG-8805 for T4 N0/1 disease with 6-year survival rate of 49%. Gandara et al. examined the feasibility of consolidation docetaxel 4–6 weeks after concurrent radiochemotherapy in patients with T4 N0/2 or T1-3 N3 disease without distant metastases NSCLC [28]. The study involved 31 patients with T4 N0/1 tumors (37%) [28]. The identical staging and eligibility criteria were used as in SWOG-9019 [9]. A N0/1 status was determined if mediastinoscopy was negative or if there was no evidence of mediastinal nodes greater than 1 cm on CT [28]. Median survival was 31 months for patients with T4 N0/1 M0 tumors in this study [28].

The present study shows overall survival rates after definitive radiochemotherapy numerically superior compared to the previous definitive radiochemotherapy studies for cT4 N0/1 M0 non-small-cell lung cancers. 5-year survival was above 50%, irrespectively whether patients were functionally, technically and prognostically non-resectable or resectable after neoadjuvant radiochemotherapy and randomly selected for definitive radiochemotherapy within the ESPATUE trial. In the prospective randomized ESPATUE phase-III trial, where resectable T4 N0/1 M0 NSCLC patients represented one third of the whole patient collective, no differences are reported between the surgical and the definitive radiochemotherapy arm overall with the limited resolution of a moderate sized randomized trial [11]. Contrary to previous studies the present study comprises a comparably long follow-up of a larger retrospective series of consecutive patients with histopathologically confirmed T4 N0/1 M0 NSCLC. At initial diagnosis patients were invasively staged by rigid bronchoscopy and mediastinoscopy or EBUS-TBNA. Furthermore, all patients underwent computed tomography and 89.1% received [^18^F]FDG-PET/CT for pre-therapeutic clinical staging. The clinical tumor stage at initial staging was reclassified according to the 8th edition of UICC using imaging studies as well as results from minimal invasive staging. 87% of tumors exhibited multiple T4-critieria. 3- and 5-year survival in the present study compares favorably by an absolute difference > 10% even to the durvalumab arm of the Pacific trial (74.9% and 57.3% compared to 56.7% [6] and 42.9% [29]). This emphasizes the more favorable prognosis of T4 N0/1 M0 patients in comparison to the broad class of stage III tumors. Cumulative incidences of distant failures at 5 years in this study were smaller than in the best arm of the conventional radiation dose arm of the RTOG 0617 trial for definitive radiochemotherapy in unresectable stage III NSCLC [26.1% (14.4%–39.4%) vs. 52.3 (45.3–58.8%)] [30], once again confirming a special role of T4 N0/1 M0 in the group of stage-III lung tumors. Important to notice is that we observed no combined distant and loco-regional failures as first site of relapse. Likewise, cumulative incidences of loco-regional failures were smaller than in the RTOG 0617 trial [15.2% (95% CI 6.6–27.2%) at 3 years in the present study vs. 49.7% (42.8–56.3%) [30]]. Kim et al. report cumulative incidences of loco-regional recurrence of 48.8% in T4 N0/1 M0 patients after definitive _cc_RTx/CTx [26]. The cumulative risk of loco-regional relapses remains significantly higher than in the trimodality treatment confirming the key role of trimodality in fit patients with resectable disease and only low risk of high-grade postoperative morbidity. However, due to other concurrent risks this does not transform into an overt survival benefit in this limited size comparison.

In the landmark trial PACIFIC by Antonia et al. evaluating consolidation immunotherapy after definitive radiochemotherapy, overall survival was enhanced by adjuvant durvalumab therapy in unresectable stage NSCLC [according to the Staging Manual in Thoracic Oncology, version 7] [6]. Though, this data may not be extrapolated directly to T4 N0/1 M0 NSCLC, in the present study 2-year survival rate of patients who received durvalumab consolidation therapy was 100% (n = 8) compared to 84.7% in the whole group supporting a potential benefit also in this subgroup of patients. Durvalumab consolidation could reduce the incidence of loco-regional and distant metastases by about one forth [6].

Expert reviews and analyses from cancer registries in Europe, USA and Korea report an increased risk of death from cardiovascular events, pulmonary diseases, and secondary malignancies in long term survivors from thoracic tumors [31–35]. Still, the majority of deaths even after 10 years of follow up is owed to cancer [31, 34, 35]. Secondary tumors are common in the long-term follow-up. The risk of the development a secondary lung cancer is reported as 1% per year [36–38]. In the present study neither distant nor local relapses of the primary tumor occurred later than 3 years. Secondary malignancies represented one of the major causes of death after 5 years of follow up and the cumulative incidences approached 15.5% (6.0–28.9%) at 7 years.

Data from the Danish Cancer Registry are supporting that resection can impact mortality far beyond the initial 30 days and that cumulative non-cancer mortality at 360 days is a valid measure for the adverse events after surgery [39]. In this study cumulative incidence of non-lung cancer related mortality was higher in patients treated with trimodality than with definitive radiochemotherapy.

## Conclusions

Long-term 5-year overall survival at 57.4% was found in patients with resectable cT4 N0/1 and non-resectable cT4 N0/1 NSCLC after induction chemotherapy followed by definitive radiochemotherapy. OS- and PFS-rates of _cc_RTx/CTx were similar within 10% difference  to the whole group of patients in this stage group treated with trimodality treatment. Loco-regional relapses were higher after definitive radiochemotherapy and non-cancer related deaths lower than with trimodality treatment. Definitive radiochemotherapy is an adequate alternative for patients with an increased risk of surgery related morbidity. Durvalumab consolidation for patients with PD-L1 expressing tumors is promising.

## Supplementary Information

**Additional file 1.** **Supplementary table 1:** standardized differences in the characteristics of the patients groups receiving definitive radiochemotherapy or trimodality treatment with or without propensity score weighting.

## Data Availability

All data generated or analyzed during this study are included in this published article.

## References

[1] Kozower BD, Larner JM, Detterbeck FC, Jones DR (2013). Special treatment issues in non-small cell lung cancer: diagnosis and management of lung cancer, 3rd ed: American College of Chest Physicians evidence-based clinical practice guidelines. Chest.

[2] Goldstraw P, Chansky K, Crowley J (2016). International association for the study of lung cancer staging and prognostic factors committee, advisory boards, and participating institutions; international association for the study of lung cancer staging and prognostic factors committee advisory boards and participating institutions. The IASLC lung cancer staging project: proposals for revision of the TNM stage groupings in the forthcoming (Eighth) edition of the TNM classification for lung cancer. J Thorac Oncol.

[3] Di Perna CA, Wood DE (2005). Surgical management of T3 and T4 lung cancer. Clin Cancer Res.

[4] Trousse D, D'Journo XB, Avaro JP (2008). Multifocal T4 non-small cell lung cancer: a subset with improved prognosis. Eur J Cardiothorac Surg.

[5] De Leyn P, Vansteenkiste J, Lievens Y, Van Raemdonck D, Nafteux P, Decker G (2009). Survival after trimodality treatment for superior sulcus and central T4 non-small cell lung cancer. J Thorac Oncol.

[6] Antonia SJ, Villegas A, Daniel D, Vicente D, Murakami S, Hui R (2018). Overall survival with durvalumab after chemoradiotherapy in stage III NSCLC. N Engl J Med.

[7] Faivre-Finn C, Vicente D, Kurata T, Planchard D, Paz-Ares L, Vansteenkiste JF, Spigel DR, Garassino MC, Reck M, Senan S, Naidoo J, Rimner A, Wu YL, Gray JE, Özgüroğlu M, Lee KH, Cho BC, Kato T, de Wit M, Newton M, Wang L, Thiyagarajah P, Antonia SJ (2021). Four-year survival with durvalumab after chemoradiotherapy in stage III NSCLC-an update from the PACIFIC trial. J Thorac Oncol.

[8] National Comprehensive Cancer Network (NCCN). NCCN Clinical Practice Guidelines in Oncology. Non-Small Cell Lung Cancer Version 6.2021—September 30, 2021, National Comprehensive Cancer Network. Available at https://www.nccn.org/professionals/physician_gls/pdf/nscl.pdf. Accessed 11.10.2021 10.6004/jnccn.2021.0058 PMC10203822 34902832

[9] Albain KS, Crowley JJ, Turrisi AT (2002). Concurrent cisplatin, etoposide, and chest radiotherapy in pathologic stage IIIB non-small-cell lung cancer: a Southwest Oncology Group phase II study, SWOG 9019. J Clin Oncol.

[10] Guberina N, Pöttgen C, Schuler M (2020). Comparison of early tumour-associated versus late deaths in patients with central or >7 cm T4 N0/1 M0 non-small-cell lung-cancer undergoing trimodal treatment: only few risks left to improve. Eur J Cancer.

[11] Eberhardt WE, Pottgen C, Gauler TC (2015). Phase III Study of surgery versus definitive concurrent chemoradiotherapy boost in patients with resectable stage IIIA(N2) and selected IIIB Non-small-cell lung cancer after induction chemotherapy and concurrent chemoradiotherapy (ESPATUE). J Clin Oncol.

[12] Verschakelen JA, Bogaert J, De Wever W (2002). Computed tomography in staging for lung cancer. Eur Respir J Suppl.

[13] Herman SJ, Winton TL, Weisbrod GL, Towers MJ, Mentzer SJ (1994). Mediastinal invasion by bronchogenic carcinoma: CT signs. Radiology.

[14] Beigelman-Aubry C, Dunet V, Brun AL (2016). CT imaging in pre-therapeutic assessment of lung cancer. Diagn Interv Imaging.

[15] Ito M, Niho S, Nihei K, Yoh K, Ohmatsu H, Ohe Y (2012). Risk factors associated with fatal pulmonary hemorrhage in locally advanced non-small cell lung cancer treated with chemoradiotherapy. BMC Cancer.

[16] Faehling M, Schumann C, Christopoulos P, Hoffknecht P, Alt J, Horn M, Eisenmann S, Schlenska-Lange A, Schütt P, Steger F, Brückl WM, Christoph DC (2020). Durvalumab after definitive chemoradiotherapy in locally advanced unresectable non-small cell lung cancer (NSCLC): real-world data on survival and safety from the German expanded-access program (EAP). Lung Cancer.

[17] Yildizeli B, Dartevelle PG, Fadel E, Mussot S, Chapelier A (2008). Results of primary surgery with T4 non-small cell lung cancer during a 25-year period in a single center: the benefit is worth the risk. Ann Thorac Surg.

[18] Schneider L, Farrokhyar F, Schieman C (2014). Pneumonectomy: the burden of death after discharge and predictors of surgical mortality. Ann Thorac Surg.

[19] van Meerbeeck JP, Kramer GW, Van Schil PE, Legrand C, Smit EF, Schramel F (2007). Randomized controlled trial of resection versus radiotherapy after induction chemotherapy in stage IIIA-N2 non-small-cell lung cancer. J Natl Cancer Inst.

[20] Li Q, Zhang P, Wang Y, Liu D, Luo L, Diasio RB (2019). T4 extension alone is more predictive of better survival than a tumour size >7 cm for resected T4N0-1M0 non-small-cell lung cancer†. Eur J Cardio-Thoracic Surg.

[21] Pöttgen C, Stuschke M, Graupner B, Theegarten D, Gauler T, Jendrossek V (2015). Prognostic model for long-term survival of locally advanced non-small-cell lung cancer patients after neoadjuvant radiochemotherapy and resection integrating clinical and histopathologic factors. BMC Cancer.

[22] Lococo F, Cesario A, Margaritora S, Dall'Armi V, Nachira D, Cusumano G (2012). Induction therapy followed by surgery for T3–T4/N0 non-small cell lung cancer: long-term results. Ann Thorac Surg.

[23] Albain KS, Rusch VW, Crowley JJ (1995). Concurrent cisplatin/etoposide plus chest radiotherapy followed by surgery for stages IIIA (N2) and IIIB non-small-cell lung cancer: mature results of Southwest Oncology Group phase II study 8805. J Clin Oncol.

[24] Stupp R, Mayer M, Kann R (2009). Neoadjuvant chemotherapy and radiotherapy followed by surgery in selected patients with stage IIIB non-small-cell lung cancer: a multicentre phase II trial. Lancet Oncol.

[25] Perentes J, Bopp S, Krueger T (2012). Impact of lung function changes after induction radiochemotherapy on resected T4 non-small cell lung cancer outcome. Ann Thorac Surg.

[26] Kim YJ, Song SY, Jeong S-Y (2015). Definitive radiotherapy with or without chemotherapy for clinical stage T4 N0–1 non-small cell lung cancer. Radiat Oncol J.

[27] Reymen B, van Baardwijk A, Wanders R, Borger J, Dingemans A-MC, Bootsma G (2014). Long-term survival of stage T4N0-1 and single station IIIA-N2 NSCLC patients treated with definitive chemo-radiotherapy using individualized isotoxic accelerated radiotherapy (INDAR). Radiother Oncol.

[28] Gandara DR, Chansky K, Albain KS (2003). Southwest Oncology Group. Consolidation docetaxel after concurrent chemoradiotherapy in stage IIIB non-small-cell lung cancer: phase II Southwest Oncology Group Study S9504. J Clin Oncol.

[29] Spigel DR, Faivre-Finn C, Gray JE (2021). Five-year survival outcomes with durvalumab after chemoradiotherapy in unresectable stage III NSCLC: an update from the PACIFIC trial. J Clin Oncol.

[30] Bradley JD, Hu C, Komaki RR (2020). Long-term results of NRG oncology RTOG 0617: standard- versus high-dose chemoradiotherapy with or without cetuximab for unresectable stage III non-small-cell lung cancer. J Clin Oncol.

[31] Janssen-Heijnen MLG, van Erning FN, De Ruysscher DK, Coebergh JWW, Groen HJM (2015). Variation in causes of death in patients with non-small cell lung cancer according to stage and time since diagnosis. Ann Oncol.

[32] Shin J, Ko H, Choi Y-H, Choi I, Song Y-M (2019). Risk of comorbid cardiovascular disease in Korean long-term cancer survivors. Eur J Cancer Care.

[33] Yoon DW, Shin DW, Cho JH, Yang JH, Jeong S-M, Han K (2019). Increased risk of coronary heart disease and stroke in lung cancer survivors: a Korean nationwide study of 20,458 patients. Lung Cancer (Amsterdam, Netherlands).

[34] Abdel-Rahman O (2017). An analysis of clinical characteristics and patient outcomes in primary mediastinal sarcomas. Expert Rev Anticancer Ther.

[35] Wu GX, Ituarte PHG, Ferrell B, Sun V, Erhunmwunsee L, Raz DJ, et al. Causes of death and hospitalization in long-term lung cancer survivors: a population-based appraisal. Clin Lung Cancer. 2020;21(3):204–213. 10.1016/j.cllc.2019.08.007. 10.1016/j.cllc.2019.08.007 31591032

[36] Johnson BE (1998). Second lung cancers in patients after treatment for an initial lung cancer. J Natl Cancer Inst.

[37] Zhou H, Shen J, Zhang Y, Huang Y, Fang W, Yang Y (2019). Risk of second primary malignancy after non-small cell lung cancer: a competing risk nomogram based on the SEER database. Ann Transl Med.

[38] Barclay ME, Lyratzopoulos G, Walter FM, Jefferies S, Peake MD, Rintoul RC (2019). Incidence of second and higher order smoking-related primary cancers following lung cancer: a population-based cohort study. Thorax.

[39] Green A, Hauge J, Iachina M (2016). The mortality after surgery in primary lung cancer: results from the Danish Lung Cancer Registr. Eur J Cardio-Thoracic Surg.

