# Supplementary material for: Long-term survival of patients with central or > 7 cm T4 N0/1 M0 non-small-cell lung cancer treated with definitive concurrent radiochemotherapy in comparison to trimodality treatment
Source: Radiat Oncol. 2022 Jul 16;17:126. doi: 10.1186/s13014-022-02080-9 (PMC9288731; doi:10.1186/s13014-022-02080-9)
Supplement: Supplementary file 1 — Additional file 1. Supplementary table 1: standardized differences in the characteristics of the patients groups receiving definitive radiochemotherapy or trimodality treatment with or without propensity score weighting. [file 13014_2022_2080_MOESM1_ESM.docx]

Supplementary table 1: standardized differences in the characteristics of the patients groups receiving definitive radiochemotherapy or trimodality treatment with or without propensity score weighting

|  | **Definitive RT/CTx** | **Trimodality treatment** | ***p*-value chi^2^ test** | **Standardized difference without PSW** | **Standardized difference with PSW** | ***p*-value chi^2^ test** |
| --- | --- | --- | --- | --- | --- | --- |
| **Age years** | | | | | | |
| < 60 years | 20 | 42 | 0.044 | -39.2% | 1.0% | 0.96 |
| ≥ 60 years | 26 | 25 |  |  |  |  |
| **Gender** | | | | | | |
| female | 12 | 16 | 0.79 | 5.1% | -10.7% | 0.57 |
| male | 34 | 51 |  |  |  |  |
| **Histology** | | | | | | |
| Adenocarcinoma | 12 | 17 | 0.033 | 1.6% | 10.9% | 0.16 |
| Squamous-cell carcinoma | 33 | 38 |  | 31.7% | 13.2% |  |
| other | 1 | 12 |  | -54.3% | -39.2% |  |
| **TNM-stage disease** | | | | | | |
| T4 N0 M0 | 34 | 53 | 0.52 | -12.3% | 7.5% | 0.70 |
| T4 N1 M0 | 12 | 14 |  |  |  |  |
| **Clinical performance status** | | | | | | |
| **ECOG-Status** | | | | | | |
| ECOG 0 | 27 | 44 | 0.40 | -14.4% | 0.2% | 0.73 |
| ECOG 1 | 18 | 23 |  | 10.0% | -2.1% |  |
| ECOG 2 | 1 | 0 |  | 21.1% | 13.6% |  |
| **NYHA-Class** | | | | | | |
| No cardiac insufficiency | 32 | 60 | 0.021 | -51.2% | -0.4% | 0.89 |
| NYHA 1 | 10 | 6 |  | 36.0% | 5.9% |  |
| NYHA 2 | 4 | 1 |  | 33.2% | -7.7% |  |
| **COPD-Score (GOLD)** | | | | | | |
| No bronchial obstruction | 17 | 31 | 0.011 | -19.0% | -7.0% | 0.63 |
| COPD 1 | 5 | 19 |  | -45.2% | 3.3% |  |
| COPD 2 | 20 | 17 |  | 38.8% | -3.5% |  |
| COPD 3 | 3 | 0 |  | 37.4% | 23.9% |  |
| COPD 4 | 1 | 0 |  | 21.1% | 13.6% |  |

Supplement table 1: PSW: propensity score weighting; preradiotherapy characteristics as possible confounders balanced by propensity score weighting. Inverse probability weighted counts are given for ordinal or dichotomous data. *P*-values were derived from a chi^2^ test. Standardized differences were calculated according to Austin PC and Mamdani MM (A comparison of propensity score methods: A case-study estimating the effectiveness of post-AMI statin use. Statistics in Medicine 2006; 25: 2084-2106).
